# Long-term cardiovascular complications following sepsis: is senescence the missing link?

**DOI:** 10.1186/s13613-021-00937-y

**Published:** 2021-12-01

**Authors:** Hamid Merdji, Valérie Schini-Kerth, Ferhat Meziani, Florence Toti

**Affiliations:** 1 INSERM (French National Institute of Health and Medical Research), UMR 1260, Regenerative Nanomedicine (RNM), CRBS (Centre de Recherche en Biomédecine de Strasbourg), FMTS (Fédération de Médecine Translationnelle de Strasbourg), University of Strasbourg, Strasbourg, France; 2 Department of Intensive Care (Service de Médecine Intensive-Réanimation), Nouvel Hôpital Civil, Hôpital Universitaire de Strasbourg, 1, place de l’Hôpital, 67091 Strasbourg Cedex, France; 3 Faculté de Pharmacie, Université de Strasbourg, Strasbourg, France

**Keywords:** Septic shock, Sepsis, Stress-induced premature senescence (SIPS), Atherosclerosis

## Abstract

Among the long-term consequences of sepsis (also termed “post-sepsis syndrome”) the increased risk of unexplained cardiovascular complications, such as myocardial infarction, acute heart failure or stroke, is one of the emerging specific health concerns. The vascular accelerated ageing also named premature senescence is a potential mechanism contributing to atherothrombosis, consequently leading to cardiovascular events. Indeed, vascular senescence-associated major adverse cardiovascular events (MACE) are a potential feature in sepsis survivors and of the elderly at cardiovascular risk. In these patients, accelerated vascular senescence could be one of the potential facilitating mechanisms. This review will focus on premature senescence in sepsis regardless of age. It will highlight and refine the potential relationships between sepsis and accelerated vascular senescence. In particular, key cellular mechanisms contributing to cardiovascular events in post-sepsis syndrome will be highlighted, and potential therapeutic strategies to reduce the cardiovascular risk will be further discussed.

## Highlights

- With improved management of patients, sepsis survivors are increasing each year.
- Early cardiovascular complications, of yet undeciphered mechanisms, are an emerging health issue in post-sepsis syndrome.
- Premature senescence of endothelium and vascular tissue is proven an accelerated process of atherogenesis in young septic rats.
- An increasing body of clinical evidence point at endothelial senescence in the initiation and development of atherosclerosis.
- Prevention of premature senescence by senotherapy and cardiological follow-up could improve long-term septic patients’ outcomes.

## Sepsis as a global health priority

Sepsis is considered as a life-threatening multiple organ dysfunction caused by a dysregulated host response to infection altering systemic arterial function [1, 2]. Although the global burden is difficult to ascertain, recent data estimated 48.9 million cases and 11 million sepsis-related deaths worldwide in 2017, which accounted for almost 20% of all global deaths [3]. Sepsis has, therefore, been recognized as a global health priority by the World Health Organization (WHO) [4]. Indeed septic shock, the most severe form of sepsis characterized by profound circulatory and cellular/metabolic failure [5], remains the leading cause of mortality in intensive care unit (ICU) [6, 7]. However, in high-income countries the long-term survival is improving, with approximately 14 million sepsis survivors each year [8], raising at the same time new health consequences and a significant burden for patients and society [9, 10]. Thus, post-sepsis syndrome involves multiple long-term deficits, including the immune, cognitive, psychiatric, renal, and cardiovascular systems [11, 12]. Notably, nearly a quarter of sepsis survivors will be readmitted to hospital within 30 days of discharge [13]. Long-term consequences greatly contribute to the high total economic cost of the disease, which is estimated to be around US$67 billion yearly in the USA alone [14].

## Cardiovascular-associated post-sepsis complications as an emerging serious health threat

Recent data suggest that the increased risk of long-term mortality among sepsis survivors could be related to increased post-sepsis cardiovascular diseases [15]. Hence, sepsis survivors have an increased risk to develop cardiovascular disease with elevated major adverse cardiovascular events (MACE), including nonfatal myocardial infarction, acute heart failure or nonfatal stroke. Hospitalization for severe pneumonia leads to an increased risk of developing cardiovascular disease that persists for at least 10 years [16]. Yende et al. found that survivors of severe sepsis had a twofold increased cardiovascular risk within the first year following hospital discharge as compared to risk- and age-matched individuals. Interestingly, in this study even the subgroup of sepsis survivors who did not have cardiovascular disease before the hospitalization, had a higher risk of subsequent cardiovascular events [17]. Recently, a meta-analysis of 27 studies (that overall included 1,950,033 sepsis survivors and 3,510,870 unique non-septic control subjects) reported that sepsis may represent a long-term cardiovascular disease risk factor, with magnitudes of relative risk comparable to those of conventional cardiovascular disease risk factors such as hypertension, dyslipidemia, and diabetes mellitus. This potential risk remaining significantly elevated for at least 5 years after hospital discharge [18]. A possible explanation would be an unusual rate of atherosclerosis of still undeciphered origin [19]. One highly likely contributor is the endothelium as demonstrated for the acute phase in preclinical data [20] and indirectly from clinical assessment of biomarkers of the endothelial dysfunction [21]. Sepsis switches the endothelial protective functions to an athero-thrombogenic profile resulting in endothelial dysfunction with altered vasoregulation, loss of barrier function, potentiating inflammation, and coagulation abnormality [22–24], finally leading to organ dysfunction.

**Fig. 1:**
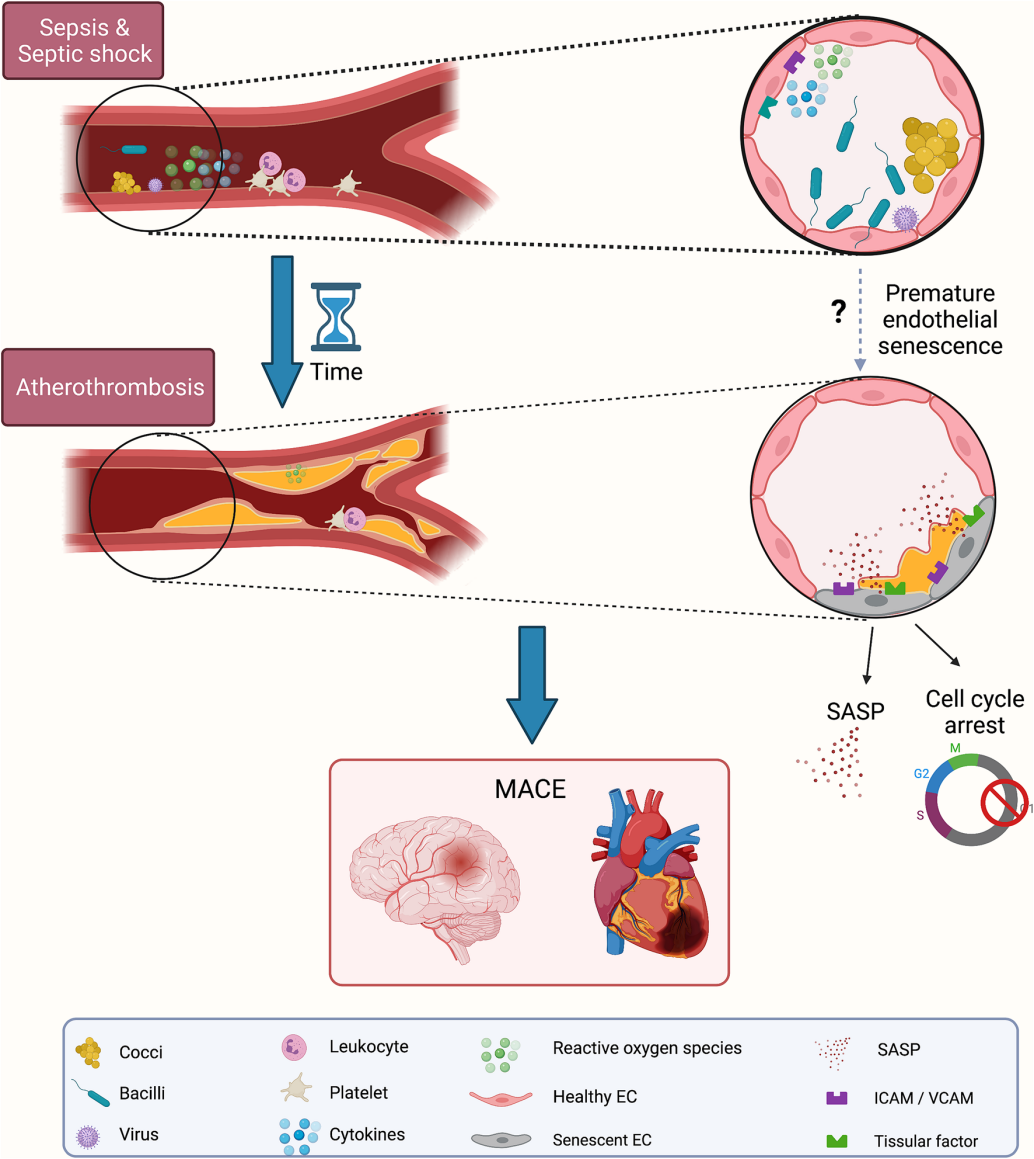
Potential mechanisms contributing to endothelial senescence-driven cardiovascular complications after sepsis and septic shock. Sepsis and septic shock survivors have an increased risk of developing cardiovascular events such as myocardial infarction and stroke. Sepsis-induced premature senescence could explain an accelerated atherogenesis process leading to early major adverse cardiovascular events. *SASP* senescence-associated secretory phenotype

A potential mechanism that may link acute and chronic endothelial dysfunction is accelerated vascular aging associated with premature endothelial senescence ultimately promoting atherothrombosis (Fig. 1).

## Vascular senescence, atherosclerosis and inflammageing

As a proof of concept of the link between endothelial senescence and atherosclerosis, a pioneer work reported that senescent endothelial cells (ECs) overlay atherosclerotic plaques, in post-mortem aortic arch histological section from patients older than 70 years. These ECs were seen as a thin continuous layer of luminal senescence-associated β-galactosidase (SA-β-Gal) activity, highly represented in vulnerable plaque [25]. In mice, early signs of endothelial senescence are detected predominantly at sites of disturbed flow and low shear stress during atherogenesis in middle-aged individuals. In senescent animal models, they are characterized by an early endothelial dysfunction, suggesting that premature ageing-related endothelial dysfunction may contribute to the focal nature of the pathology and possibly also to its initiation and progression [26]. In rodent models or human samples, a progressive expression of senescence biomarkers p53, p21, p16 and accumulating SA-β-Gal activity occur in ageing vascular tissues, including endothelial cells, vascular smooth muscle cells, and macrophages [27–32].

In an experimental model of atherosclerosis-prone mice, Kaynar and colleagues [33] corroborated the association between sepsis and the occurrence of cardiovascular events by showing that the cecal ligation and puncture (CLP) accelerates aortic atherosclerotic plaque formation within the subsequent 5 months. Although these data confirm the association between sepsis and atherosclerosis, the authors concluded that the mechanism underlying this accelerated atherogenesis remains to be fully elucidated. Indeed, these data point at the need to develop long-term follow-up murine models of sepsis.

Recently, our team has provided new insights by characterizing a premature vascular senescence in rats after CLP surgery [34]. Sepsis was found to accelerate premature senescence in the aorta tissue with a significant upregulation of p53 and downstream p21 and p16 senescence markers as early as 7 days after CLP, values peaking 3 months later. Of note, p53 was mainly detected in the aortic endothelium by immunofluorescence and confocal microscopy, thereby confirming its prime and key role. In addition, our data suggest a link between arterial senescence and a remote endothelial dysfunction in conductance and resistance arteries that was characterized by long-term blunted endothelium-dependent relaxation and contraction at 3 months.

One of the other main contributors to the link between sepsis, senescence and atherosclerosis for cardiovascular disease is “inflammageing” [35]. Inflammageing is a condition characterized by high blood and tissue levels of pro-inflammatory markers associated with susceptibility to cardiovascular diseases in the elderly. The physiopathology of inflammageing remains poorly deciphered to date and relies on immune cell dysregulation, microbiota alteration, increased intestinal permeability, chronic infections, and central obesity. At the cellular level, mitochondrial-mediated oxidative stress, activation of the NLRP3 inflammasome, and genetic susceptibility contribute to inflammageing as well as the pro-inflammatory senescence-associated secretory phenotype (SASP) [36]. Advanced atherosclerotic plaques exhibit both senescence markers such as p16 and the SASP which further fuels inflammation, thereby destabilizing the atherosclerotic plaque, suggesting a key contribution of inflammageing [37].

## Senescence: causative or coincidental to ageing?

Physicians and philosophers of ancient Greece have already questioned aging as a disease or a natural process [38]. The Hippocratic Corpus asserted that old age inevitably led to frailty and then death and therefore, considered aging an incurable disease. The interrogation persisted in the Latin world, *“Senectus ipsa morbus est”,* reflecting the disease paradigm while the Roman Galen asserted that, unlike diseases that are abnormal, ageing is universal and is, therefore, a natural process. Although the answer is not yet conclusive and this dichotomy still persists nowadays, recent progress in biology allows a better understanding of aging and senescence [39].

### Replicative senescence: a reversible biological clock?

Senescence describes a state of permanent replicative arrest in normally proliferative cells, losing their ability to divide. Senescence is not equivalent to quiescence or death. Indeed, senescent cells remain alive and metabolically active for a long period of time [40]. Besides exiting the cell cycle, the senescent state is accompanied by a failure to re-enter the cell cycle in response to mitogenic stimuli. Other signatures of senescence are a metabolic reprogramming, autophagy and abnormal chromatin rearrangement such as heterochromatin foci, also named senescence-associated heterochromatic foci (SAHF) whereupon proliferation-related genes are silenced. In addition, the senescence-associated secretory phenotype (SASP) initiates a paracrine dissemination of an oxidative and pro-inflammatory signal. At the level of the organism, senescence may appear as a defense mechanism that limits the replication of old or damaged cells bearing accumulated DNA repair errors and therefore preserves the homeostatic balance.

“Replicative senescence” is considered a biological clock triggered by aging. It is caused by a progressive shortening of telomeres upon each cell division. Described in 1961, the “Hayflick limit” was the first in vitro observation of a limited human fibroblast proliferation capacity, their mitosis being abolished after 50 cell divisions, despite the addition of growth factors and the absence of contact inhibition [41]. Initially, several investigators were skeptical, claiming an isolated in vitro artifact. There is now accumulating in vivo evidences that senescence is a true biological response [42] progressively occurring in age-related pathologies, including type 2 diabetes, obesity, atherosclerosis, chronic obstructive pulmonary disease (COPD), pulmonary fibrosis, and many others diseases [43]. In the recent decades, the improvement of public health has extended the human lifespan thereby favoring senescence as a major emerging contributing factor to chronic diseases in the elderly [44].

### Accelerated senescence: a stress-induced ageing

In the year 2000, pioneering work by Olivier Toussaint and others showed that there is another major way, other than chronological aging, for cells to become senescent. Indeed, a significant cellular stress can trigger senescence even in young cells through a phenomenon known as stress-induced premature senescence (SIPS) [45]. Recent studies suggest that sepsis, during which many stressors are severely and significantly exacerbated, is a condition of accelerated senescence.

## Features of senescent cells shared by replicative senescence cells and stress-induced premature senescence cells

Several markers are used to detect senescent cells, among which senescence-associated beta-galactosidase (SA-β-Gal) activity is the current gold standard for the detection of senescence in vitro [46]. The characteristic elevation of the β-Gal activity in senescent cells is the consequence of both the enzyme upregulation [47] and an increase in the lysosomal mass [48] with paradoxical decline of their degradative ability. β-Galactosidase strictly operates at pH 4.5 in healthy cells while it is still active at a pH of 6 in senescent cells, thereby enabling the quantification of a senescence-associated β-galactosidase (SA-β-Gal) activity [49], one of the first markers to be used [50].

However, SA-β-Gal activity measurement is a comparative assessment. In vivo*,* its high sensitivity to sampling and storage conditions and the need of a non-senescent control make the analysis challenging. Nevertheless, key characteristics in all types of cell senescence are the cell cycle arrest and the upregulation of p53, p21 and p16, often used as alternate markers. Still, cell cycle arrest itself cannot be considered a truly surrogate marker of senescence, since multiple other cellular responses can drive a stable replicative arrest. Indeed, the inability to express proliferation genes, even in a promitogenic environment [51, 52] distinguishes senescence from quiescence, a non-proliferative state of the cells that is readily reversed in response to mitogens. Of note, mTOR plays a key role in the shift between senescence or quiescence: when both p53 and mTOR are activated, cells become senescent, while the sole activation of p53 leads to quiescence [53].

In the absence of reliable direct assessment, several nonexclusive markers are reported in the literature to monitor cell senescence. The shift to a SASP [54], also termed senescence-messaging secretome [55], is undoubtedly the most characteristic and relevant feature of senescent cells and a potential biomarker. SASP is associated with the secretion of a plethora of immune modulators, inflammatory cytokines, growth factors, chemokines, and proteases in the close microenvironment of senescent cells.

Each cell linage is characterized by a specific SASP pattern of secreted molecules, several studies suggesting up to 103 molecules per cell type [43], often determined by the initiator of the senescence response [56]. Key components are pro-inflammatory tumor necrosis factor alpha (TNF-α), cytokines interleukin-6 (IL-6), interleukin-8 (IL-8), and interleukin-1 alpha (IL-1 α) having a juxtacrine role on the surrounding cells, and matrix metalloproteinases (MMP-1 and -3) acting on the remodeling of the extracellular matrix [57]. SASP relies on pro-inflammatory signaling pathways including NF-κB, mTOR and p38 mitogen-activated protein kinase (MAPK) [58].

### How is senescence different from apoptosis?

**Fig. 2:**
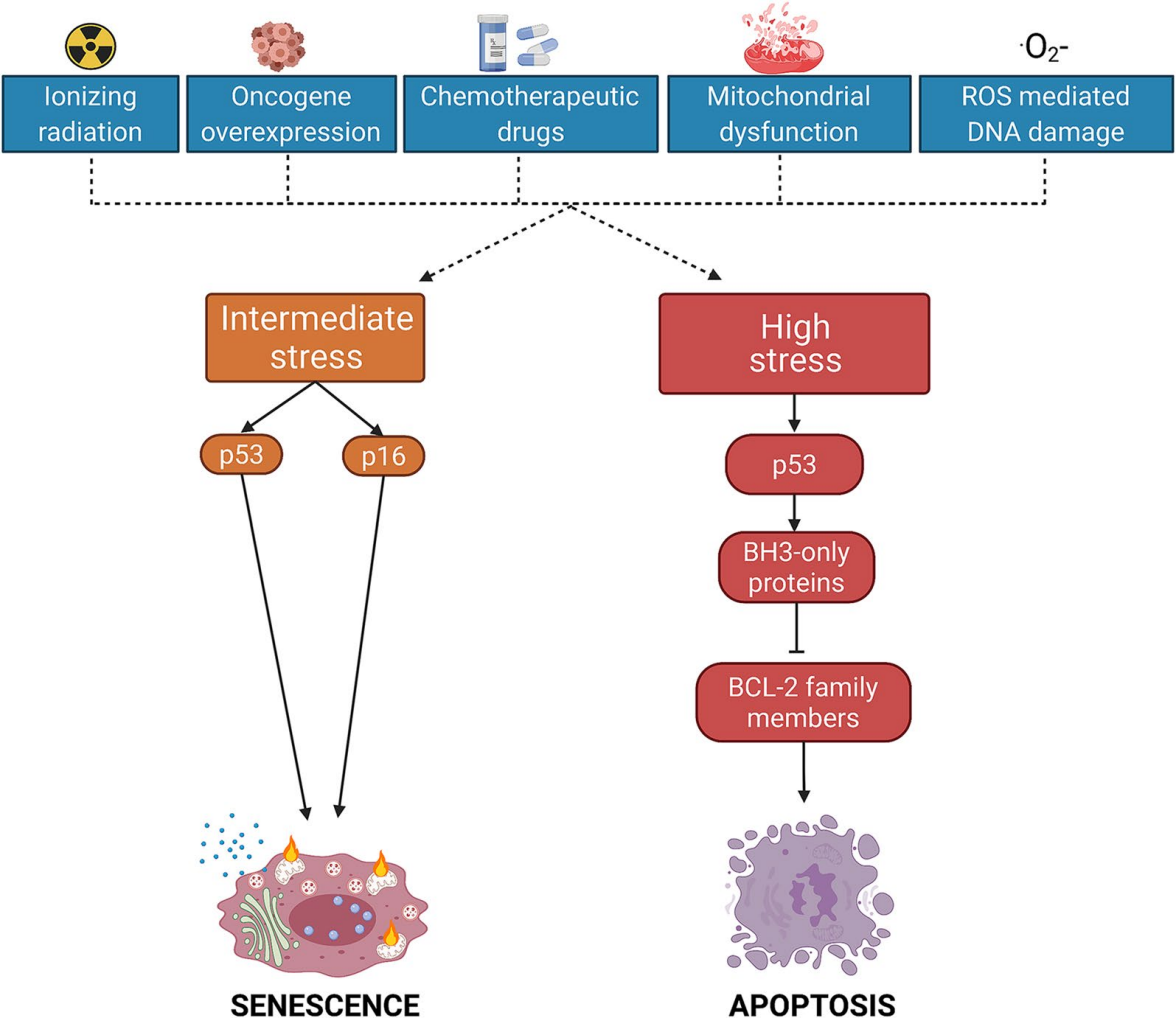
Difference between senescence and apoptosis. Intermediate stress can lead to senescence via p53 and p16 pathway, resulting in persisting cell dysfunction. High cellular stress can induce apoptosis through upregulation of p53, resulting to cell death and elimination. High level of p53 contributes to the induction of BH3-only proteins (BIM, PUMA, NOXA) that inhibits pro-survival BCL-2 family members (BCL-XL, MCL-1, BCL 2)

Apoptosis and senescence pathways drive alternative cell fates that can often be triggered by the same stressors. Indeed, once cells enter senescence, they become resistant to extrinsic apoptosis by overexpressing decoy receptor 2 (DCR2) [43] and to intrinsic apoptosis [59–61] at least in part via the upregulation of BCL-2 family members [62], being themselves under the eventual control of p53, a transcription factor involved in autophagy, DDR, cell cycle progression and apoptosis [63]. While high stress can lead to apoptosis, then cell death and elimination, intermediate stress can lead to senescence with persisting cell dysfunction (Fig. 2).

### Endothelial senescence and vascular ageing

**Fig. 3:**
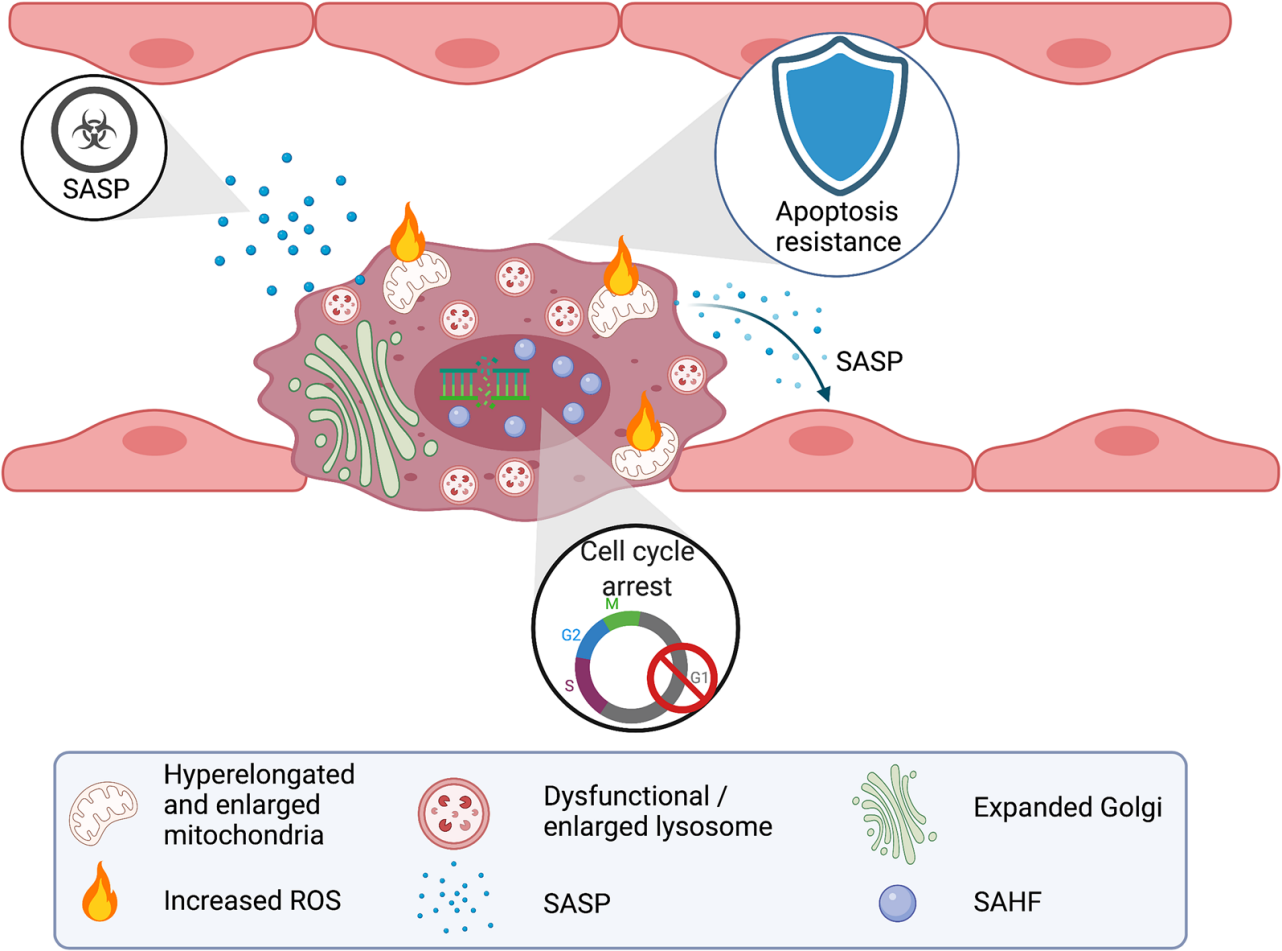
Characteristics of senescent endothelial cell. Senescent cells become irregular and flat with cytoplasmic and nuclear enlargement, multiple organelle modifications, including enlarged and dysfunctional lysosomes enclosing lipid and protein aggregates. Senescent cells can exhibit hyperelongated mitochondria resulting from unbalanced mitochondrial fission and fusion thereby favoring ROS generation. An expanded Golgi apparatus is also observed, along with nuclear enlargement and chromatin condensation such as SAHF. Senescence-associated dysfunction includes the SASP with autocrine and paracrine effects, the apoptosis resistance and cell cycle arrest. *ROS* reactive oxygen species, *SAHF* senescence-associated heterochromatin foci, *SASP* senescence-associated secretory phenotype

Endothelial senescence is associated with morphological and metabolic changes. The EC becomes flatter (“egg on a plate morphology”) and enlarged with an increasingly polypoid nucleus (Fig. 3). Such changes are accompanied by a loss of cytoskeleton integrity, and altered cell proliferation, migration and angiogenesis [64]. Senescent ECs show decreased endothelial nitric oxide (NO) production, increased endothelin-1 (ET-1) release, elevated inflammatory response [65], and have a specific SASP profile detailed in Table 1 [66–69].

**Table 1:** Main endothelial SASP components

Main endothelial SASP components	
Pro-inflammatory mediators	TNF-α, TGF-β, IL-1, IL-6, CSFs
Pro-inflammatory chemokines	CXCL-1, CXCL-8, CCL-2
Proteases and mediators of tissue remodeling	MMPs, PAI-1
Growth factors	VEGF, EGF, IGFBPs

*TNF-α* tumor necrosis factor alpha, *TGF-β* transforming growth factor beta, *IL-1* interleukin-1, *IL-6* interleukin-6, *CSFs* colony-stimulating factor, *CXCL-1* chemokine (C-X-C motif) ligand-1, *CXCL-8* chemokine (C-X-C motif) ligand-8, *CCL-2* C-C motif chemokine ligand 2, *MMPs* matrix metalloproteinases, *PAI-1* plasminogen activator inhibitor-1, *VEGF* vascular endothelial growth factor, *EGF* epidermal growth factor, *IGFBPs* insulin-like growth factor-binding protein

**Fig. 4:**
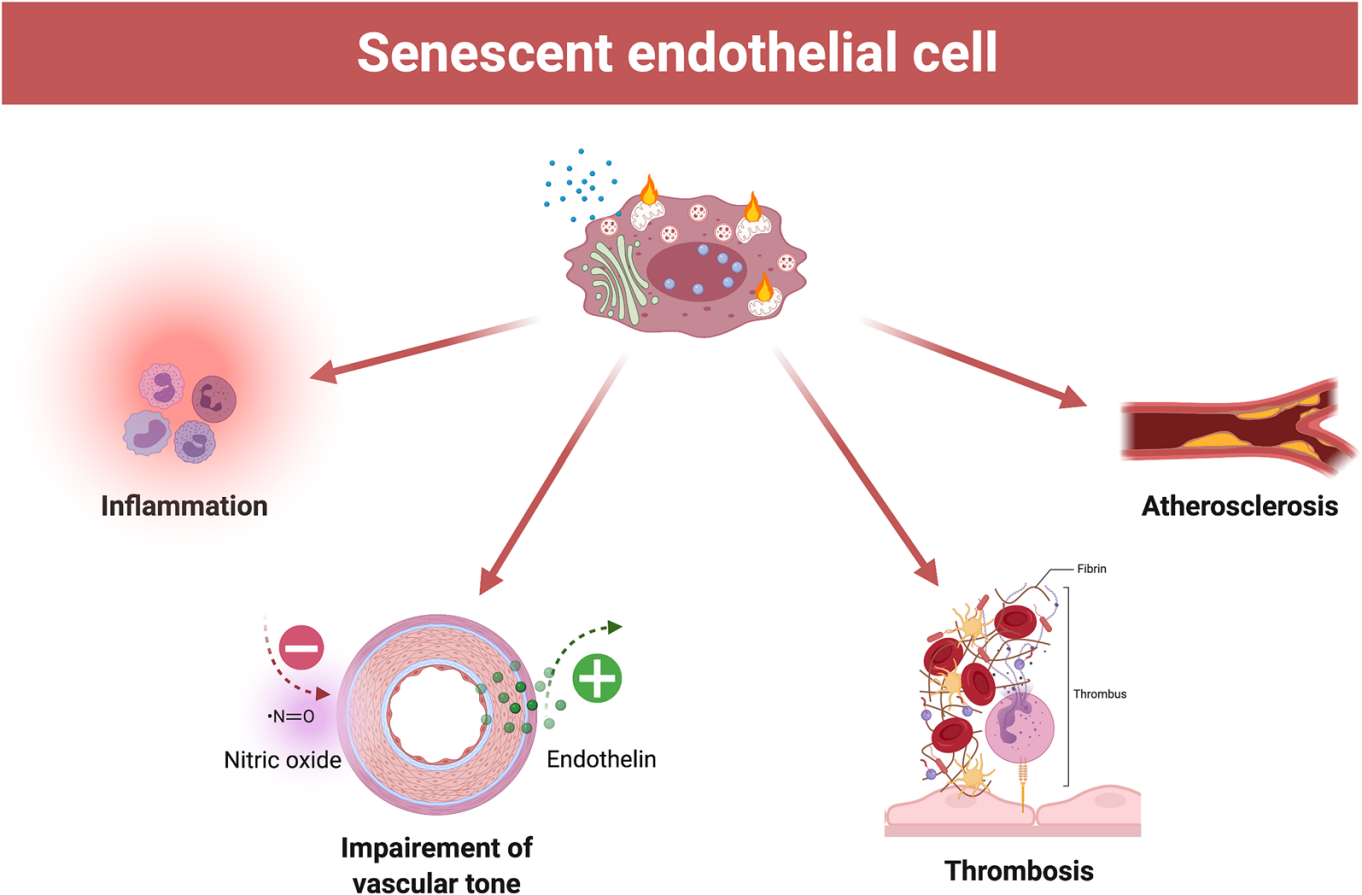
Features of dysfunctional senescent endothelial cell. Accumulation of senescent endothelial cells impedes vascular homeostasis. Main consequences include a progressive acquisition of an inflammatory endothelial phenotype, a procoagulant state, a proatherogenic phenotype, and the loss of vascular tone with reduced NO availability and increased release of endothelin. *NO* nitric oxide

Accumulating senescent ECs induce vascular, structural, and functional changes shifting the endothelium from a protective monolayer preserving physiological vascular tone to a pro-inflammatory, athero-thrombogenic dysfunctional barrier, all of which favor cardiovascular disease [70, 71] (Fig. 4).

## What are the paths leading to cellular senescence?

One of the major discoveries of the early twenty-first century is that in addition to replicative senescence, cells can also undergo unplanned senescence when subjected to stressors. SIPS and replicative senescence share overlapping pathways with distinct checkpoints.

**Fig. 5:**
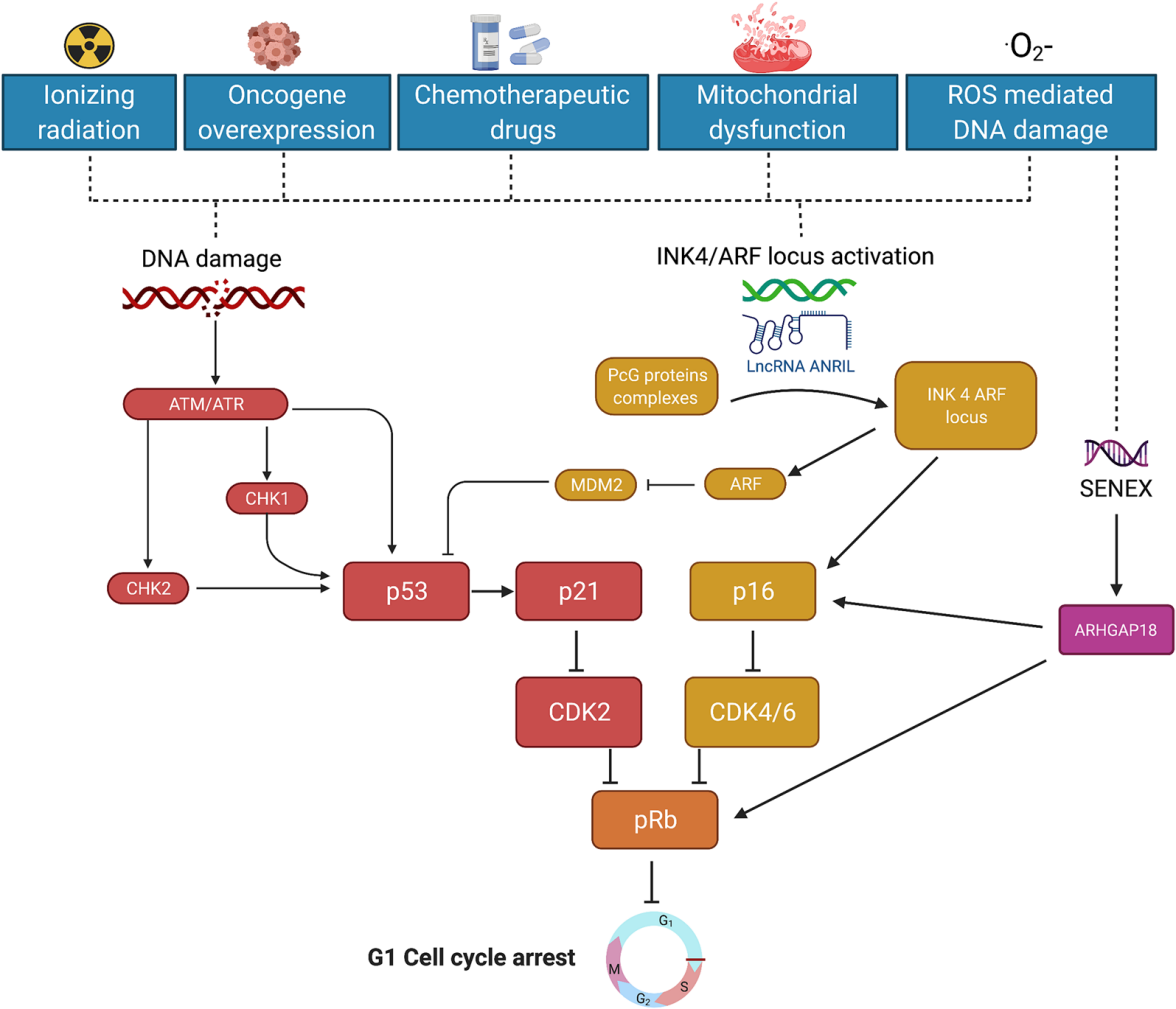
Main pathways leading to cellular senescence. Mechanisms that drive cellular senescence include the direct activation of the DNA damage response (DDR) through the ATM/ATR pathway and/or of the INK4a/ARF locus through the assembly of PcG protein complexes eventually via the ANRIL scaffolding Lnc RNA. The INK4 family, among which p16, are cyclin-dependent kinase inhibitors targeting CDK4/6. Ultimately, p53/p21 and p16/Rb pathways are key players driving senescence. *ANRIL*: antisense non-coding RNA in the INK4 locus, *ARF* ADP ribosylation factor, *ARHGAP18* (Rho GTPase activating protein 18), *ATM* ataxia-telangiectasia mutated, *ATR* ataxia-telangiectasia mutated and Rad3 related, *CDKs* cyclin-dependent kinases, *Chk1* checkpoint kinase 1, *Chk2* checkpoint kinase 2, *DDR* DNA damage response, *INK4* inhibitors of CDK4, *p16/Rb* p16/retinoblastoma protein, *PcG* polycomb, *Lnc* *RNA* long non-coding RNA, *ROS* reactive oxygen species

DNA damage response (DDR) is a major driver in both replicative senescence and SIPS, respectively, initiated by telomere shortening or different stressors (Fig. 5). In replicative senescence, telomeres via the telosome complex [72], prevent the DDR machinery from recognizing chromosome-free ends as double-strand breaks to be repaired, a potential threat leading to erroneous chromosome recombination or fusion events [73]. When telomeres become critically short, the protective telosome is no longer recruited to the DDR, thereby favoring senescence. The other senescence pathway, triggered by stressors, is controlled by the INK4/ARF locus, extensively studied in oncogene-induced senescence (OIS) [74].

p53 is the main checkpoint in the DDR pathway. It can be activated directly by ATM/ATR or indirectly via the activation of checkpoint kinase 1 (Chk1) and checkpoint kinase 2 (Chk2), two serine/threonine-specific protein kinases (Fig. 5).

In SIPS, p53 can be activated via ARF (ADP ribosylation factor), a small GTPase of the RAS superfamily family, which blocks the activity of MDM2, an ubiquitin ligase leading to p53 degradation. p53 induces the transcription of the downstream cyclin-dependent kinase inhibitor p21, which blocks CDK2 activity, resulting in hypophosphorylated retinoblastoma protein (pRB). The binding of hypophosphorylated pRB to the transcription factor E2F will further suppress the expression of S‑phase genes leading to a cell cycle arrest [75].

The activation of the INK4/ARF locus not only triggers ARF, but also p16, a member of the INK4 cell cycle inhibitors. p16 directly binds to the cyclin-dependent kinases CDK4 and CDK6, thereby blocking the downstream phosphorylation of the pRB tumor suppressor.

Ongoing investigations on the activation of the INK4/ARF locus point at the formation of the polycomb group (PcG) proteins complexes (PRC 1 and PRC 2) as the main initiator of the response to stressors. PcG proteins act as transcriptional repressors through the trimethylation or mono-ubiquitination of histones H3 and H2A, thereby controlling the expression of genes involved in DNA repair at specific.

How is PcG altered during senescence is yet not completely understood, recent data pointing at a possible implication of silencing miRNA [76]. Strikingly, in various cell models of senescence, the interaction of PcG with the INK4/ARF locus appears also under epigenetic control via long non-coding (Lnc) RNAs serving as scaffolds, such as ANRIL (antisense non-coding RNA in the INK4 locus) [77–79].

Amplification of the senescent response occurs through heterochromatinization of cell-cycle genes in SAHF (Fig. 3) [80] and via the SASP-driven production of pro-inflammatory cytokines such as IL-6 that favor the cell-cycle arrest [81].

### Stress induces premature senescence: SIPS

In 2000, Toussaint and colleagues reported a pioneer observation that cultured human fibroblasts robustly entered a senescence-like state several days after repeated exposure to mild treatment with *tert*-butylhydroperoxide with sublethal oxidative stress [82]. This work was then corroborated using sustained or repeated cell treatments by numerous chemical stressors like ethanol [83], chronic exposure to pollutants (cigarette smoke) or irradiation (UV-B light) [84]. SIPS is mainly initiated by DNA damage, DNA breaks activating the DDR pathway in the absence of telomere shortening [85].

Recent data have challenged the concept that SIPS is a telomere-independent process, distinct from replicative senescence. Indeed, DNA damage during SIPS occurs randomly all over the genome including telomeres. However, whereas most of the DNA damage will be repaired within 24 h, telomeric regions will remain unrepaired for months, maintaining a sustained unresolved DNA damage [86]. Of note, this reveals that pathways leading to senescence, either premature or replicative, may at some point share intricate features. Recently, SENEX, an endothelial senescence-inducing gene, discovered as a result of serendipity, acting in response to H_2_O_2_ was shown to induce the p16/Rb pathway by up-regulating both p16 mRNA and protein together with a decrease in the hyperphosphorylated Rb protein level [87]. This gene does not alter the expression of either p53 or p21 nor affects telomere length pointing at a prevailing p16 pathway (Fig. 5).

## Sepsis as a stress factor inducing premature senescence in several tissues

While pathophysiological mechanisms of sepsis are widely described in the elderly [88], this review will focus on sepsis-induced premature senescence.

Indeed, during the previous decade, several in vitro and in vivo studies have highlighted the association between sepsis and premature senescence (Table 2). In vitro, a single 24-h exposure to lipopolysaccharide (LPS) induces the senescence of type II pulmonary alveolar epithelial cells detectable after 7 days by SA-β-Gal activity with no telomere shortening [89]. Viruses are also septic stressors of pulmonary cells leading to elevated SA-β-Gal activity [90]. As evidenced in human pneumocyte type II cells (A549*)* and nasopharyngeal cells (HEp-2)*,* the human respiratory syncytial virus (hRSV) causes strong ATM/p53/p21-dependent activation of the DDR, as well as the nuclear recruitment of phosphorylated γ-H2AX, a typical marker of the DDR response. Same effect has been demonstrated in murine Neuro2a cells on which the Avian H7N9 influenza virus induces cellular senescence in vitro [91]. Premature LPS-induced senescence has been also characterized in murine BV2 microglia cells [92], in adipocytes progenitors [93] or dental pulp stem cells [94].

**Table 2:** Studies of sepsis-induced senescence in cell, preclinical and clinical studies

Cells	Species of origin		Sepsis model		Analysis time points		Results		References	
In vitro										
Microglia cells (BV2 cell)	C57BL/6 mouse		10 ng/ml LPS stimulation: once 3 times: once every 48 h for 4 h each 6 times: once every 24 h for 4 h each		After 1, 6 or 12 days		Kinetics (6–12 days): •Inhibition of cell proliferation •Elevated degree of: − Cell cycle arrest in the G0/G1 phase − The aging associated proteins p53 − Senescence-associated β-galactosidase activity − Senescence-associated heterochromatic foci (SAHF)		[92]	
Type II pulmonary alveolar epithelial cells (A549 immortalized cells)	Human		5—20 μg/ml LPS single stimulation for 24 h		After 1, 3 or 7 days		•Elevated degree of: − Senescence-associated β-galactosidase activity •No decrease in telomere length		[89]	
Dental pulp stem cells (DPSCs)	Human		10 ng/ml E*scherichia coli* LPS (serotype 0111:B4) stimulation: once for 6 h 3 times: once every 48 h for 6 h each 6 times: once every 24 h for 6 h each		After 1 h		Senescence-like morphology •Inhibition of cell proliferation •Elevated degree of: − Cell cycle arrest in the G1 phase − Senescence-associated β-galactosidase activity − The aging associated p16^INK4A^ − of p16^INK4A^ mRNA *•Knockdown of p16*^*INK4A*^ *expression by siRNA transfection reversed the senescent features of LPS-treated DPSCs*		[94]	
Adipocyte progenitors (stromal-vascular cells)	C57BL/6 mouse		0.2 μg/ml LPS stimulation for 24 h		After 3 days		•Elevated degree of: − p53 phosphorylation − Senescence-associated β-galactosidase activity − β-galactosidase-positive cells − mRNA indicating significant SAPS (TNFα, IL-1β, IL-6, monocyte chemoattractant protein-1 (MCP-1), and VEGFα) •*No accelerate telomere shortening*		[93]	
Type II pulmonary alveolar epithelial cells (A549 immortalized cells) Human nasopharyngeal cells (HEp-2 immortalized cells)	Human		Human respiratory syncytial virus (*Pneumovirus* genus of the *Paramyxoviridae family)*		After 48 h		•Senescence-associated secretory phenotype (SASP) in supernatant •Elevated degree of: − The aging associated proteins p53 − Senescence-associated β-galactosidase activity		[90]	
Neuroblastoma Neuro2a Cells	Mouse		H7N9 *Influenza *A Virus		After 3 days		•Senescent cell-like morphology •Increase senescence-associated β-galactosidase activity		[91]	
In vivo										
Blood, spleen and kidney samples (unspecified cell type)	Young male BALB/c mice		15 mg/kg LPS intraperitoneal injection		After 1 h or 48 h		•Dose-dependent telomere shortening in the spleen and liver at 48 h (but not at 1 h) measured by quantitative polymerase chain reaction (PCR) •No difference in telomerase expression in kidney homogenates 1 h after LPS		[95]	
Lung tissue (unspecified cell type)	Young male C57BL/6 mice		Two-hit mouse model using CLP followed by sublethal *Pseudomonas aeruginosa* lung infection 4 h later		24 h after *Pseudomonas aeruginosa* lung infection		•Upregulation of: − Senescence-associated biomarker p16^ink4a^ − Senescence-associated β-galactosidase activity		[96]	
Vascular tissue	Young Wistar male rats		CLP		3, 7 or 90 days after CLP		•Upregulation of: − The aging associated proteins p53, p21 and p16 − The aging associated proteins p53 localized in the endothelium		[34]	

In vivo, data confirming sepsis-induced premature senescence in young individuals are scarce. In a murine endotoxemia model a ~ 20% reduction in telomere length by qPCR was reported in spleen and kidney, 48 h after intraperitoneal injection of a high LPS concentration, while no other senescence marker was assessed [95]. More in-depth characterization was brought by elevated p16 and SA-β-Gal activity in lung tissue measured after 24 h in a two-hit septic mice model using CLP followed by sublethal *Pseudomonas aeruginosa* infection [96]. Additionally, airway epithelium senescence was also evidenced by γ-H2AX and CDKN2A labeling from day 4 to day 30 in hRSV-infected mice [90]. Same effect has been demonstrated in murine Neuro2a cells in vitro on which the H7N9 influenza virus induces cellular senescence.

In a rat model, we recently evidenced that CLP-induced sepsis causes a time-dependent arterial accumulation of senescence markers, peaking at 3 months post-induction and associated with vascular dysfunction [34].

To date, the only data describing accelerated senescence after sepsis in human were reported by Oliveira et al*.* Their analysis showed that telomere length, from blood samples of patients who developed sepsis in the trauma department, was significantly shortened 1 week after sepsis initiation [95].

Altogether, these observations strongly suggest that a senescent shift may progressively occur after sepsis as an ongoing process thereby questioning the timescale to study consecutive tissue damages.

## Next-generation therapies targeting senescent cells for post-sepsis cardiovascular disorders

**Fig. 6:**
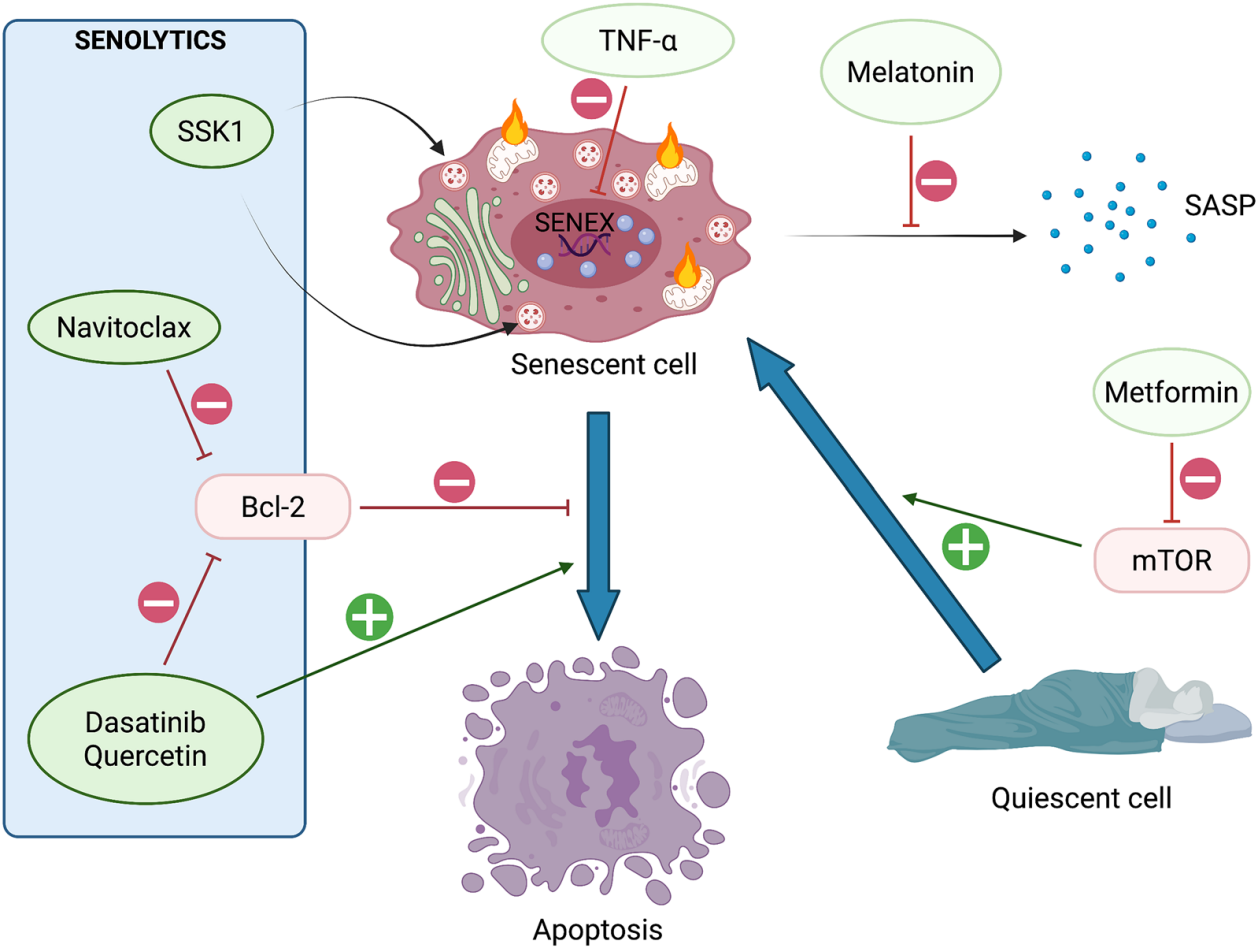
Main senotherapeutic drug targets. Senolytics aiming to eliminate senescent cells favor downstream apoptosis or directly target senescent lysosomes (SSK1). Senostatics preventing the acquisition of a senescent state limit the conversion of quiescent cells, the progressive acquisition of SASP and the inhibition of SENEX. *SASP* senescence-associated secretory phenotype, *SSK1* senescence-specific killing compound 1

Many pharmacological studies have indicated that specifically eliminating senescent cells (“senolysis”) by using senolytic drugs or by suppressing the senescent phenotype with senostatics may contribute to reversal of the aging phenotype (Fig. 6) [97, 98] and should be considered as a next-generation therapy for atherosclerotic disorders [99, 100]. These senotherapies are usually non-specific and do target multiple pathways.

### Senolytics

In a major 2018 study, Kirkland and his colleagues at the Mayo Clinic provided a proof-of-concept evidence that transplanted senescent cells can cause physical disability and reduced lifespan in young and middle-aged mice. They also demonstrated that intermittent oral administration of a senolytic cocktail of dasatinib and quercetin significantly reversed the effect of senescent cells and increased median survival by 36% [98].

To date, one main senolytic strategy is to shift the senescent cells into apoptotic ones by triggering the member of the BCL-2 family [101], most of them being up-regulated in senescent cells [102]. Indeed, the most studied senolytics are dasatinib (a pan inhibitor of tyrosine kinases), quercetin (a flavonoid present in many fruits and vegetables with antioxidant and anti-inflammatory properties, mainly targeting PI3-kinase and serpins) and navitoclax previously named ABT263 (a mimetic of the BH-3 domain of anti-apoptotic proteins BCL-2 and Bcl-xL) [62, 103, 104]. Navitoclax would appear promising in the prevention of potential sepsis-induced cardiovascular disorders, since it was demonstrated to efficiently reduced plaque burden, number and average size in atherosclerosis-prone mice with established senescence [99]. Similarly, dasatinib and quercetin were shown to prevent vasomotor dysfunction in aged mice and reduce senescence burden and arterial plaque calcification in an ApoE^−^/^−^ high-fat diet murine model [105]. While targeting BCL-2 may lead to unwanted cellular triggering and toxicity [106], senescence-specific killing compound 1 (SSK1) would better target senescent cells with low impact on the self-renewal of target cells. This new senolytic prodrug is specifically cleaved by the enhanced lysosomal β-galactosidase activity characterizing senescent cells and transformed into cytotoxic gemcitabine inducing apoptosis, as demonstrated in mice and human ECs in vitro [107].

In 2019, first evidence that senolytics (dasatinib and quercetin) are safe and efficient in humans was published [108]. Later the same year, the same team from the Mayo clinic reported for the first time in human that these senolytics reduced key circulating SASP factors (IL and MMP), but also senescence markers (p21, p16 and SA-β-Gal activity) in adipose tissue biopsies [103].

### Senostatics

Inhibiting SASP, via melanin for example [109], without causing adverse effects is challenging because many pathways that may activate SASP (such as NF-κB or mTOR) are also involved in critical processes such as tumor surveillance or the immune system [110].

Interestingly, SENEX is a TNFα-sensitive gene and in vitro treatment by low concentration of TNFα prompts the endothelial downregulation of this gene leading to apoptosis, confirming SENEX as a promising target in the early prevention of sepsis-induced endothelial senescence [87].

Another vascular protective strategy would be to prevent the shift from endothelial quiescence to senescence by inhibiting the mTOR pathway. Confirmation was brought in atheroprone ApoE^−^/^−^ adult mice treated by metformin that inhibited endothelial cell senescence and thus contributed to partially decreased atherosclerotic plaque formation [111]. This is of particular interest because metformin is also known to exert protective effect on endothelial cells in sepsis via adenosine monophosphate-activated protein kinase AMPK activation (which exert inhibition of mTOR) [112].

## Conclusion

Post-septic cardiovascular disease, as a part of the morbidity and mortality observed in the post-sepsis syndrome, is one of the emerging health issues. Premature senescence of endothelium and vascular tissue appears to be one of the mechanisms involved in the accelerated atherogenesis in sepsis survivors. Targeting pro-senescent endothelial cells with senotherapy in sepsis seems promising to delay endothelial senescence and improve vascular health and long-term outcomes after sepsis.

## Data Availability

Not applicable.

## Abbreviations

ATM: Ataxia-telangiectasia mutated
ATR: Ataxia-telangiectasia mutated and Rad3 related
Chk: Checkpoint kinase
CLP: Cecal ligation and puncture
COPD: Chronic obstructive pulmonary disease
DCR2: Decoy receptor 2
DDR: DNA damage response
DNA: Deoxyribonucleic acid
EC: Endothelial cell
ET-1: Endothelin-1
hRSV: Human respiratory syncytial virus
ICU: Intensive care unit
IL: Interleukin
*Ldlr*^−/−^: Low-density lipoprotein receptor-deficient
LPS: Lipopolysaccharide
MACE: Major adverse cardiovascular events
MMP: Matrix metalloproteinase
NO: Nitric oxide
PI3K: Phosphoinositide 3-kinase
pRB: Retinoblastoma protein
ROS: Reactive oxygen species
SA-β-Gal: Senescence-associated β-galactosidase
SAHF: Senescence-associated heterochromatic foci
SASP: Senescence-associated secretory phenotype
SIPS: Stress-induced premature senescence
SSK: Senescence-specific killing compound
US$: United States dollar
USA: United States of America
UV: Ultraviolet
VIS: Virus-induced senescence
WHO: World health organization

## Glossary

Apoptosis: is a form of programmed cell death with a key role in the removal of potentially harmful and damaged cells such as precancerous or virus-infected cells. Apoptotic cells are characterized by DNA fragmentation, membrane blebbing, formation of apoptotic bodies, and activation of proteolytic enzymes such as caspases.
Atherosclerosis: a chronic inflammatory disease of large and medium-sized arteries that lead to the formation of fibrofatty lesions in the artery wall predominantly at sites of disturbed flow where endothelial senescence emerges.
Cecal ligation and puncture (CLP): rat model of sepsis. CLP-rats undergo a laparotomy, a ligation and puncture of the cecum, which is then reintegrated in the peritoneum. Rats develop peritonitis within few hours, resulting in sepsis or septic shock. The severity of sepsis can be modulated via the number and size of the punctures.
DNA damage response (DDR): involves a complex network of genes that can promote cell-cycle arrest to repair DNA lesions induced by different kind of stress. DDR can induce cell senescence in case of irreparable DNA damage.
Endothelium: monolayer-type of epithelium lining the interior of the heart and blood vessels. Under normal circumstance, the endothelial surface is a protective barrier which displays antiaggregant, anticoagulant and anti-inflammatory features.
H_2_O_2_: Hydrogen peroxide is part of the reactive oxygen species, a group of molecules produced in the cell through metabolism of oxygen. It is one major contributor to oxidative damage.
Inflammageing: a condition that progressively develops with age and characterized by modification of the immune system and elevated levels of blood inflammatory markers that favor high susceptibility to chronic morbidity, invalidity, frailty, and premature death.
Lipopolysaccharide (LPS): essential component of the outer membrane of Gram-negative bacteria. Frequently used to mimic the initial acute inflammatory response to sepsis, both in vivo and ex vivo.
Major adverse cardiovascular events: is a composite endpoint frequently used in cardiovascular research. First defined as a composite of nonfatal myocardial infarction, nonfatal stroke, and cardiovascular death (classical 3-point MACE). It can also include hospitalization for heart failure in some studies (4-P MACE). Detection and treatment of the risk factors for MACE are critical to improve health and longevity.
Post-sepsis syndrome: Consists of immunological, cardiovascular, and cognitive deficits persisting long after hospital discharge, resulting in more frequent rehospitalizations due to recurrent sepsis, altered quality of life, and increased morbidity and mortality. It affects up to 50% of sepsis survivors.
Senescence-associated heterochromatic foci (SAHF): are specialized domains of facultative heterochromatin contributing to silencing of proliferation-promoting genes (such as E2F target genes) in senescent cells.
Senescence associated secretory phenotype (SASP): defines the ability of senescent cells to express and secrete a broad range of extracellular modulators including cytokines, chemokines, proteases, growth factors and lipids. SASP can mediate tumor suppression and wound healing but also chronic inflammation and age-related diseases.
Sepsis: dysregulated host response to an infection, resulting in life-threatening organ dysfunction.
Septic shock: sepsis with acute circulatory failure, defined by low blood pressure requiring vasopressors and by hyperlactatemia, reflecting tissue hypoxia.
Telomere: specific DNA–protein structures found at both ends of each chromosome, protecting genome from nucleolytic degradation, unnecessary recombination, repair, and interchromosomal fusion. With replication cycles telomeres grow shorter or dysfunctional that could lead to DNA damage response.
Telosome: consists of telomere-specific proteins involved in the protection of telomere, preventing from degradation and activation of unwanted repair systems. Also named “the shelterin complex”, it plays a crucial role in replicative senescence and ageing-related pathologies.
